# Shaping the future of fiber technology: exploring functional and smart innovations

**DOI:** 10.1093/nsr/nwae373

**Published:** 2024-10-21

**Authors:** Meifang Zhu

**Affiliations:** State Key Laboratory for Modification of Chemical Fibers and Polymer Materials, College of Materials Science and Engineering, Donghua University, China

On 14 February 1928, American chemist Dr. Wallace Hume Carothers wrote a letter to Dr. John R. Johnson at Cornell University, in which he articulated, for the first time, his deep insights into polymerization and polymeric molecules [1]. These pioneering ideas directly led to the invention and commercialization of nylon by DuPont in the 1930s, marking the dawn of the chemical fiber era. Over nearly 100 years of development, chemical fibers have evolved from a singular category into a diverse range of materials utilized in fields spanning clothing and home textiles to aerospace and electronic devices [2]. With superior properties such as high strength, corrosion resistance and lightweight characteristics, chemical fibers not only satisfy everyday needs but also play a crucial role in high-tech applications, revolutionizing various industries.

The continuous innovation in new fiber materials has catalyzed the emergence of functional and smart fibers, driving a technological transformation in the fiber industry and creating new opportunities across multiple sectors [3–5]. Therefore, we have organized a special topic on ‘Functional and Smart Fibers’ in *National Science Review*. This special topic aims to explore the latest advancements in functional and smart fibers, with the goal of highlighting innovative research on the design, synthesis, fabrication and application of fibers that can significantly enhance smart technologies, improve energy efficiency and promote environmental sustainability, among other benefits. By presenting cutting-edge studies in the field, this special topic seeks to underscore the vital role that fibers play in advancing technologies that tackle urgent global challenges.

In 1954, Professors Baojun Qian and Bairong Fang at the East China Institute of Textile Science and Technology (now Donghua University) established China's first chemical fiber program. In 1994, the College of Materials Science and Engineering at Donghua University was founded. Over the course of 70 and 30 years of development, respectively, we have made outstanding contributions to the transformation and upgrading of China's chemical fiber industry, which produces 70% of the world's output, and have achieved the localization of strategic fiber materials such as viscose-based carbon fiber and aramid. Furthermore, we have cultivated a substantial number of talented professionals in the field of fibers and materials. This special topic also commemorates the 30th anniversary of the College of Materials Science and Engineering and the 70th anniversary of the chemical fibers program (now the discipline of Materials Science and Engineering) at Donghua University.

This special topic comprises 13 papers, including 1 research highlight, 3 perspectives, 4 research articles, 4 reviews and 1 interview. In the first research highlight [6], Professor John Ballato from Clemson University shares his insights on novel high-quality semiconductor fibers fabricated through convergent thermal drawing, as well as their unique applications in smart wearable optical sensing, based on remarkable work recently published in *Nature* [7]. The first perspective [8], contributed by Professor Fabien Sorn from École Polytechnique Fédérale de Lausanne, analyzes recent advances and potentials in both fundamental research and industrial applications of thermally drawn multi-material fibers. The second perspective [9], from Professors Meifang Zhu and Wei Yan and Dr. Hailiang Wang at Donghua University, reviews the development history, current status and future research directions of fibers, including chemical, functional and smart fibers. The third perspective [10], contributed by Professors Meifang Zhu, Yinjun Chen and Junrong Yu at Donghua University, discusses the past, present and future of high-performance fibers.

The first research article [11], by Professors K. Jimmy Hsia and Huajian Gao at Nanyang Technological University, focuses on novel shape memory polymer fibrillar adhesives with exceptional performance for high-payload applications. The second research article [12], by Professors Liangbing Hu and Teng Li at the University of Maryland, develops strong, stiff and humidity-responsive smart cellulosic fibers derived from abundant natural grass. The third article [13], by Professor Shengjie Peng at Nanjing University of Aeronautics and Astronautics, introduces Fe-N co-doped carbon nanofibers for water activation-induced oxygen reduction reactions. The fourth research article [14], by Professor Lei Wei at Nanyang Technological University and Professor Wei Yan at Donghua University, presents high-capacity Li–S fiber batteries for wearable energy storage.

In the review section, the first article [15] by Professor Yuanlong Shao at Peking University reviews the dispersion, processing and properties of carbon nanotube fibers. The second review article [16], from Professor Ray H. Baughman at the University of Texas at Dallas and Professor Jiuke Mu at Tianjin University, examines innovations in electrically powered artificial muscle fibers. The third review article [17], by Professor Xiaoting Jia at Virginia Tech, covers the development and future directions of multi-material fibers for modern biomedical applications. The fourth review article [18], from Professor Yi Cui at Stanford University and Professor Yucan Peng at Peking University, discusses thermal management using innovative fibers and textiles. In the interview [19], Professor Stephen Cheng at South China University of Technology shares his insightful thoughts on the current state of polymer science and fibers, their impact and the future core challenges in the field.

We hope this special topic can serve as a valuable resource for researchers, engineers and professionals working in the fields of functional and smart fibers. The collective efforts presented here underscore the diversity and potential of fiber-based technologies to address some of the most pressing challenges in modern society, including aerospace, energy, environmental sustainability, healthcare and smart wearables. By showcasing groundbreaking research from global leaders in fiber science and engineering, we aim to inspire future advancements that push the boundaries of what fibers can achieve. Finally, we would like to extend our heartfelt gratitude to the editorial team of *National Science Review*. Their dedication and professionalism have been instrumental in bringing this special topic to fruition.

## Notes

Guest Editor of Special Topic and Editorial Board Member of NSR
